# Glucose-albumin ratio (GAR) as a novel biomarker of postoperative urinary tract infection in elderly hip fracture patients

**DOI:** 10.3389/fmed.2024.1366012

**Published:** 2024-07-15

**Authors:** Wei Wang, Wanyun Tang, Wei Yao, Qiaomei Lv, Wenbo Ding

**Affiliations:** ^1^Department of Orthopedics, Dandong Central Hospital, China Medical University, Dandong, China; ^2^Department of Oncology, Dandong Central Hospital, China Medical University, Dandong, China

**Keywords:** hip fracture, elderly, postoperative urinary tract infection, prognosis, glucose-albumin ratio

## Abstract

**Purpose:**

Postoperative urinary tract infections (UTIs) worsen the prognosis of elderly patients with hip fractures. This study aimed to assess the predictive ability of blood-based biomarkers, specifically the glucose-albumin ratio (GAR), in predicting postoperative UTIs.

**Methods:**

A retrospective observational study of 1,231 patients from a Level I trauma center was conducted. We evaluated the prognostic and predictive value of 15 biomarkers, including the glucose-albumin ratio, in elderly patients with hip fractures. The primary outcome measure was the incidence of postoperative UTIs.

**Results:**

The glucose to albumin ratio transformed into GAR was superior to any other biomarker in predicting postoperative UTIs in elderly hip fracture patients (AUC = 0.756, *p* < 0.001). Elevated GAR (using the best cut-off value of 0.18) was independently associated with postoperative UTIs (OR 3.20, 95% CI 2.23–4.58). Further analysis dividing GAR levels into four groups according to quartiles showed that compared to patients with GAR levels of Q1 (< 0.14), GAR levels of Q2 (0.14–0.17; OR 2.11, 95% CI 1.07–4.15), Q3 (0.17–0.21; OR 3.36, 95% CI 1.74–6.52) and Q4 (> 0.21; OR 7.55, 95% CI 3.84–14.83) patients had significantly higher odds of UTIs.

**Conclusion:**

GAR holds potential as a novel biomarker for predicting postoperative UTIs in elderly patients with hip fractures.

## Introduction

The global incidence of hip fractures is on the rise, with projections stating a dramatic increase from 1.31 million cases in 1990 to an estimated 6.26 million by 2050 (1–4). This escalating prevalence places significant pressure on healthcare systems while leading to an increase in postoperative complications and mortality rates (5–8). Efficient surgical intervention and diligent postoperative management have proved effective for hip fracture patients (4, 9). Furthermore, postoperative urinary tract infections (UTIs) in elderly patients with hip fractures present significant healthcare concerns, often not given due attention (10–13). Significantly, the routine application of indwelling urinary catheters in orthopedic wards can intensify the adverse effects associated with hip fractures, leading to increased patient discomfort and unfavorable prognosis (12, 14–16). Hence, rapid identification of hip fracture patients prone to UTI is paramount through certain indicators.

Post-traumatic hyperglycemia commonly observed in elderly patients upon admission with hip fractures is a contributing factor to the occurrence of UTIs (17, 18). While it is widely recognized that diabetic patients are at an increased risk of urinary tract infections, the association between stress-induced hyperglycemia upon admission due to trauma and the risk of urinary tract infections remains unclear (19–21). In addition to investigating blood glucose levels, attention must also be directed toward serum albumin as a significant indicator of nutritional status (22, 23). Hypoalbuminemia (serum albumin < 35 g/L), a manifestation of malnutrition commonly observed in elderly hip fracture patients, is intrinsically associated with postoperative complications and premature mortality (24, 25). Its development is influenced by several factors, particularly chronic disease, inflammatory response, and insulin resistance (22, 26). Hypoalbuminemia exacerbates insufficient nutritional intake, hindering recovery and increasing infection risk (27).

The existing literature suggests an association between post-traumatic hyperglycemia, hypoalbuminemia, and increased susceptibility to infection, but well-designed studies directly linking these factors to the risk of UTIs are limited (18, 28, 29). This gap underscores the clinical importance of the proposed study to explore the link between hyperglycemia, hypoalbuminemia, and the occurrence of UTIs in elderly hip fracture patients. With the aforementioned considerations, the objective of this paper is to examine the potential of the glucose-albumin ratio (GAR) as a novel biomarker for predicting postoperative UTIs in elderly patients with hip fractures, thereby contributing to preoperative risk assessment and postoperative nutritional intervention strategies to improve surgical outcomes and providing valuable insights to improve patient prognosis.

## Materials and methods

### Study design and data sources

We conducted a retrospective analysis of elderly patients with hip fractures who were admitted to our institution between March 2013 and June 2023. The study was approved by the Dandong Central Hospital Ethics Committees (No. DDZX-20230711), and informed consent was waived. Our study cohort comprises patients undergoing surgical treatment for hip fractures, all of whom underwent dual diagnostic evaluation with preoperative X-ray imaging and three-dimensional CT reconstruction. However, patients meeting any of the following criteria were excluded: (1) age below 60 years; (2) presence of old fractures, pathologic fractures, multiple fractures, periprosthetic fractures, or open fractures; (3) previous conservative treatment, revision surgery, reoperation, or long-term use of immunosuppressive agents, such as glucocorticoids; (4) preoperative diagnosis of urinary tract infections; (5) lack of urinary culture during hospitalization or laboratory tests, including urinalysis; (6) in-hospital mortality or incomplete information available for any reason.

### Variables

The demographic data collected for analysis included age, gender, smoking status, and alcoholism. Past comorbidities considered were hypertension, diabetes, cardiovascular disease, stroke, chronic kidney disease, prostatic hyperplasia, urolithiasis, vesicoureteral disease, and a history of neoplasms. The baseline health information of these patients was routinely collected and recorded in the hospital’s electronic system by nursing staff upon the patients’ admission to our medical institution. Additionally, the data encompassed the type of hip fracture, classification based on the American Society of Anesthesiologists (ASA), surgery method, the utilization of indwelling catheterization, duration of indwelling catheterization, duration of the surgery, and length of bed rest. In our study, the calculation of patient bed rest duration began upon admission and ended when patients were capable of bearing weight and standing post-surgery.

### Laboratory indicators

Blood samples were collected from patients with hip fractures within 24 h of admission to obtain relevant biomarker data. The biomarkers collected included: blood glucose levels (mmol/L), albumin levels (g/L), D-dimer levels (μg/L), alanine aminotransferase levels (IU/L), high-density lipoprotein levels (mmol/L), erythrocyte count (× 10^12^/L), leukocyte count (× 10^9^/L), neutrophil count (× 10^9^/L), lymphocyte count (× 10^9^/L), platelet count (× 10^9^/L), mean platelet volume (fL), red blood cell distribution width (%), aspartate aminotransferase levels (IU/L), cholesterol levels (mmol/L), low-density lipoprotein levels (mmol/L), triglyceride levels (mmol/L), hemoglobin levels (g/L), creatinine levels (μmol/L), uric acid levels (μmol/L), and blood urea nitrogen levels (mmol/L).

### Definition of outcome

The primary outcome measured in this study was the incidence of postoperative UTIs. To exclude preadmission urinary tract infection in patients with hip fractures, urine analysis and culture results upon admission were analyzed. After undergoing orthopedic surgical treatment, routine urine cultures were performed every three days (on Tuesdays and Fridays). To ensure standardized urine collection, clinical nurses in the orthopedic unit received regular training from the hospital’s infection committee on aseptic techniques, standard disinfection methods, and prompt sample delivery to the microbiology laboratory. In accordance with the guidelines from the Centers for Disease Control and Prevention (30), a UTI was defined if patients met the following criteria: (1) fever (> 38 degrees Celsius or 100.4 degrees Fahrenheit), urgency, frequency, urethral pain, suprapubic pressure, and lumbar and dorsal horn pain or tenderness; and (2) a positive urine culture (bacteriuria > 10^5^ CFU/mL) or positive urinalysis results (leukocyte esterase and nitrite in mid-stream urine specimens). Prior to the commencement of the study, three researchers underwent standardized training in systematic operation and interpretation of UTIs indicators (WY, WW, and WT). The determination of infection events was independently performed by three members, with any disagreements being resolved by senior researchers (QL and WD). Finally, patients diagnosed with UTIs routinely received antibiotic treatment.

### Statistical analysis

Continuous variables were reported as median ± interquartile range (IQR), while categorical variables were presented as absolute numbers and percentages. Statistical significance was defined as two-sided *P*-values < 0.05. Five blood-based biomarkers were identified and tested. Two of these biomarkers (blood glucose and D-dimer) exhibited an increase in adverse outcomes, while the remaining three biomarkers (alanine aminotransferase, albumin, and high-density lipoprotein) showed a decrease in adverse outcomes. All these factors were employed to identify the combination (glucose-albumin ratio) that yielded the highest predictive accuracy for postoperative UTIs in patients with hip fractures.

Receiver operating characteristic (ROC) curves and their corresponding area under the curve (AUC) were calculated to evaluate the predictive performance of different biomarkers for postoperative UTIs. To ascertain the optimal cutoff, we employed the Youden index, computed as the sum of sensitivity and specificity minus one. We systematically derived Youden index values by calculating corresponding sensitivity and specificity at various points along the ROC curve. The optimal cutoff was identified as the point on the ROC curve that maximizes the Youden index.

The covariates were analyzed using multivariate logistic regression to calculate the odds ratio (OR) and corresponding 95% confidence interval (CI). Variables with a *p*-value < 0.10 in the univariate regression were included in the multivariate logistic regression analysis. The covariates included in the logistic regression models were selected based on previous studies (31, 32) and clinical expertise. E-values were employed to evaluate the strength of observed associations considering potential unmeasured confounding (33). These e-values were computed utilizing an online e-value calculator available at https://www.evalue-calculator.com/evalue/. Moreover, patients were categorized into four groups (Q1: < 0.14, Q2: 0.14–0.17, Q3: 0.17–0.21, and Q4: > 0.21) based on the quartile distribution of the target biomarkers, aiming to enhance the assessment of the dose-effect relationship with postoperative UTIs. Lastly, propensity score matching was conducted as a sensitivity analysis (34). In our PSM 1:1 matching process, we utilized the nearest neighbor matching algorithm to match the high-level blood sugar or albumin group with the normal group, with a caliper value set at 0.1 standard deviation. We employed the standardized mean difference (SMD) to assess any imbalance between the two groups, considering SMD ≥ 0.10 as indicative of imbalance. Statistical analyses were performed using R software version 4.0.3.

## Results

**FIGURE 1:**
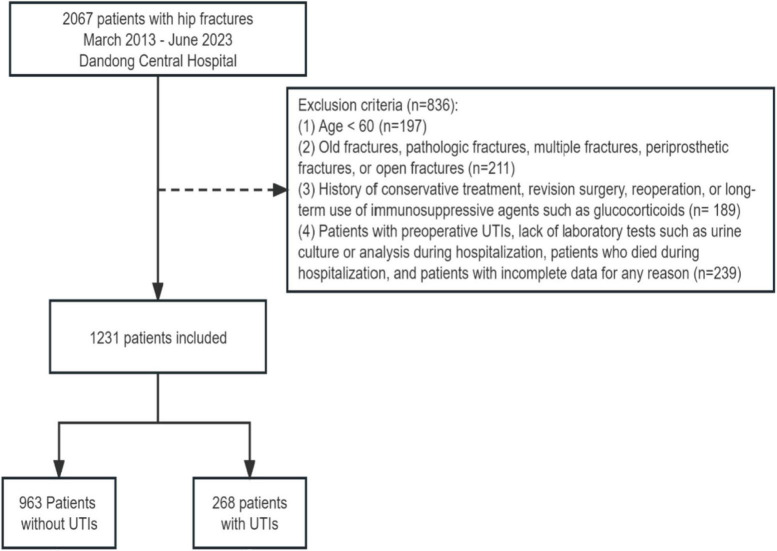
Flow diagram of patients included in the cohort.

We initially identified 2,067 patients with hip fractures occurring between March 2013 and June 2023. Following a rigorous exclusion process, a total of 836 patients were excluded from the study. Specifically, patients were excluded if they were under 60 years old (*n* = 197), presented with old fractures, pathologic fractures, multiple fractures, periprosthetic fractures, or open fractures (*n* = 211), had a history of conservative treatment, revision surgery, reoperation, or long-term use of immunosuppressive agents such as glucocorticoids (*n* = 189), or if they had preoperative urinary tract infections (UTIs), lacked laboratory tests such as urine culture or analysis during hospitalization, died during hospitalization, or had incomplete data for any reason (*n* = 239). After applying these exclusion criteria, a final cohort of 1,231 elderly patients with hip fractures was included in the study. Among them, 268 patients (21.77%) developed postoperative UTIs (Figure 1). Table 1 presents the baseline characteristics of elderly patients with hip fractures, including a mean age of 74.99 years and 741 (60.19%) female patients. Patients in the UTIs group were significantly older (*p* < 0.001), had a higher proportion of females (*p* < 0.001), a higher prevalence of indwelling urinary catheters and longer catheterization duration (*p* < 0.001), a longer period of bedridden status (*p* < 0.001), a higher prevalence of comorbidities, and a higher preoperative ASA grading (*p* < 0.01) compared to those in the non-UTIs group. Furthermore, patients with intertrochanteric fractures exhibited a higher incidence of UTIs compared to patients with femoral neck and proximal femur fractures (*p* < 0.001). Likewise, patients who underwent intramedullary nail fixation showed a higher incidence of UTIs than those who underwent alternative surgical approaches (*p* < 0.001).

**TABLE 1:** Baseline characteristics of the 1,231 patients with hip fractures.

Characteristics	Total (*n* = 1,231)	UTIs development		
		Non-UTIs (*n* = 963)	UTIs (*n* = 268)	*p*-value
**Demographic**				
Age, × years (Mean ± SD)	74.99 ± 9.47	74.05 ± 9.42	78.38 ± 8.90	< 0.001
Female gender (*n*, %)	741 (60.19%)	541 (56.18%)	200 (74.63%)	< 0.001
Smoking (*n*, %)	213 (17.30%)	181 (18.80%)	32 (11.94%)	0.01
Alcohol (*n*, %)	142 (11.54%)	123 (12.77%)	19 (7.09%)	0.01
**Comorbidities**				
Hypertension (*n*, %)	625 (50.77%)	449 (46.63%)	176 (65.67%)	< 0.001
Diabetes (*n*, %)	285 (23.15%)	176 (18.28%)	109 (40.67%)	< 0.001
Cardiovascular disease (*n*, %)	378 (30.71%)	272 (28.25%)	106 (39.55%)	< 0.001
Stroke (*n*, %)	326 (26.48%)	238 (24.71%)	88 (32.84%)	0.01
Chronic kidney disease (*n*, %)	64 (5.20%)	33 (3.43%)	31 (11.57%)	< 0.001
Prostate hyperplasia (*n*, %)	26 (2.11%)	16 (1.66%)	10 (3.73%)	0.04
Urolithiasis (*n*, %)	19 (1.54%)	11 (1.14%)	8 (2.99%)	0.03
Vesicoureteral disease (*n*, %)	57 (4.63%)	28 (2.91%)	29 (10.82%)	< 0.001
Neoplasms (*n*, %)	112 (9.10%)	94 (9.76%)	18 (6.72%)	0.13
**Operation**				
**Fracture type**				
Femoral neck fracture (*n*, %)	639 (51.91%)	542 (56.28%)	97 (36.19%)	< 0.001
Intertrochanteric fracture (*n*, %)	521 (42.32%)	369 (38.32%)	152 (56.72%)	
Subtrochanteric fracture (*n*, %)	71 (5.77%)	52 (5.40%)	19 (7.09%)	
**ASA classification**				
III-IV (*n*, %)	700 (56.86%)	526 (54.62%)	174 (64.93%)	0.003
I-II (*n*, %)	531 (43.14%)	437 (45.38%)	94 (35.07%)	
**Surgery method**				
Total hip arthroplasty (*n*, %)	153 (12.43%)	122 (12.67%)	31 (11.57%)	< 0.001
Hemiarthroplasty (*n*, %)	311 (25.26%)	251 (26.06%)	60 (22.39%)	
Intramedullary nail fixation (*n*, %)	413 (33.55%)	289 (30.01%)	124 (46.27%)	
Internal fixation with steel plate (*n*, %)	167 (13.57%)	122 (12.67%)	45 (16.79%)	
Internal fixation with hollow nails (*n*, %)	187 (15.19%)	179 (18.59%)	8 (2.99%)	
Catheterization (*n*, %)	570 (46.30%)	400 (41.54%)	170 (63.43%)	< 0.001
Indwelling catheter time, × days (Mean ± SD)	1.79 ± 3.47	1.27 ± 2.28	3.69 ± 5.66	< 0.001
Intraoperative time, × hours (Mean ± SD)	1.66 ± 0.81	1.64 ± 0.78	1.74 ± 0.90	0.08
Bedridden time, × days (Mean ± SD)	5.89 ± 4.04	5.60 ± 3.86	6.93 ± 4.49	< 0.001
**Laboratory findings**				
GLU, mmol/L (Mean ± SD)	6.97 ± 2.78	6.51 ± 2.21	8.65 ± 3.79	< 0.001
ALB, g/L (Mean ± SD)	37.90 ± 4.74	38.40 ± 4.63	36.10 ± 4.67	< 0.001
D-dimer, ug/L (Mean ± SD)	5.03 ± 5.12	4.61 ± 4.86	6.54 ± 5.72	< 0.001
ALT, IU/L (Mean ± SD)	21.01 ± 42.05	22.19 ± 47.18	16.76 ± 10.06	0.06
HDL, mmol/L (Mean ± SD)	1.26 ± 0.43	1.28 ± 0.44	1.17 ± 0.37	< 0.001

UTIs, Urinary tract infections; ASA, The American Society of Anesthesiologists Physical Status Classification System; GLU, glucose; ALB, albumin; ALT, alanine aminotransferase; HDL, high-density lipoprotein.

**FIGURE 2:**
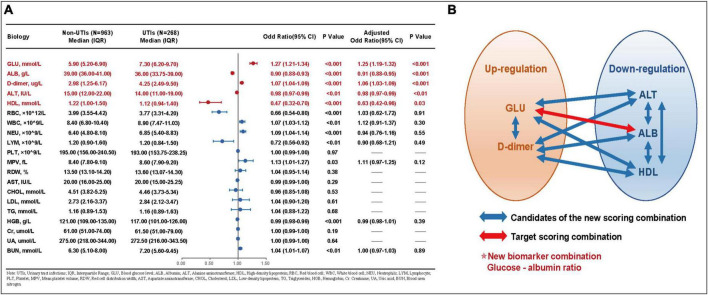
**(A)** Baseline data and univariate and multivariate analysis for each laboratory factors. **(B)** Schematic chart for the combination of laboratory factors in this study. UTIs, urinary tract infections; IQR, interquartile range; GLU, blood glucose lever; ALB, albumin; ALT, alanine aminotransferase; HDL, high-density lipoprotein; RBC, red blood cell; WBC, white blood cell; NEU, neutrophils; LYM, lymphocyte; PLT, platelet; MPV, mean platelet value; RDW, red cell distribution width; AST, aspartate aminotransferase; CHOL, cholesterol; LDL, low-density lipoprotein; TG, triglycerides; HGB, hemoglobin; Cr, creatinine; UA, uric acid; BUN, blood urea nitrogen.

**FIGURE 3:**
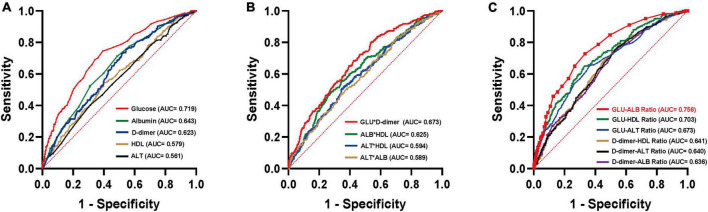
ROC curves analysis to evaluate the predictive value of each combination for postoperative UTIs in patients with hip fractures: GLU-ALB Ratio **(C)** showed the highest accuracy for the prediction of postoperative UTIs compared with established scores including other laboratory factors **(A,B)** in hip fracture patients. ROC, receiver operating characteristic; AUC, area under the curve.

The collected biomarkers and their baseline levels are presented in Figure 2A. Subsequently, after a screening process, 5 blood biomarkers and 10 potential combinations were selected for further investigation, as depicted in Figure 2B. Out of these combinations, the prediction of postoperative UTIs in elderly hip fracture patients was most accurately achieved by the GAR (AUC = 0.756, Figure 3C), surpassing both individual biomarkers and their combinations (Figure 3). Significantly, the GAR exhibited superior accuracy compared to other biomarkers (*p* < 0.001, Table 2). The optimal cut-off value for the GAR was determined to be 0.18. Additionally, the Data Supplementary Material enclosed in Supplementary Figures 1, 2 summarizes the predictive capabilities of individual biomarkers for postoperative UTIs.

**TABLE 2:** Assessment of the characteristic parameters of each biomarker.

Variables	AUC (95% CI)	ACC (%, 95% CI)	SEN (%, 95% CI)	SPE (%, 95% CI)	PPV (%, 95% CI)	NPV (%, 95% CI)	DeLong test (*p*-value)
GLU-ALB Ratio	0.756 (0.725–0.788)	68.0 (68.0–68.0)	72.8 (67.4–78.1)	66.7 (63.7–69.6)	37.8 (33.6–42.0)	89.8 (87.6–92.0)	Reference^&^
GLU-ALT Ratio	0.673 (0.636–0.710)	64.0 (64.0–64.0)	65.3 (59.6–71.0)	63.7 (60.6–66.7)	33.3 (29.3–37.4)	86.8 (84.3–89.3)	< 0.001
GLU-HDL Ratio	0.703 (0.668–0.738)	66.5 (66.4–66.5)	64.9 (59.2–70.6)	66.9 (63.9–69.8)	35.3 (31.1–39.5)	87.3 (84.9–89.7)	< 0.001
D-dimer-ALT Ratio	0.640 (0.604–0.676)	52.3 (52.3–52.4)	77.2 (72.2–82.3)	45.4 (42.2–48.5)	28.2 (25.0–31.5)	87.8 (84.9–90.6)	< 0.001
D-dimer-ALB Ratio	0.636 (0.600–0.672)	54.0 (54.0–54.1)	73.1 (67.8–78.4)	48.7 (45.5–51.9)	28.4 (25.0–31.8)	86.7 (83.8–89.6)	< 0.001
D-dimer-HDL Ratio	0.641 (0.605–0.677)	54.7 (54.6–54.7)	74.3 (69.0–79.5)	49.2 (46.1–52.4)	28.9 (25.5–32.3)	87.3 (84.5–90.1)	< 0.001
GLU*D-dimer	0.673 (0.638–0.709)	53.5 (53.5–53.6)	79.9 (75.0–84.7)	46.2 (43.1–49.4)	29.2 (25.9–32.5)	89.2 (86.5–91.9)	< 0.001
ALT*ALB	0.589 (0.551–0.627)	61.7 (61.6–61.7)	47.4 (41.4–53.4)	65.6 (62.6–68.6)	27.7 (23.6–31.8)	81.8 (79.0–84.5)	< 0.001
ALT*HDL	0.594 (0.556–0.632)	62.0 (61.9–62.0)	50.7 (44.8–56.7)	65.1 (62.1–68.1)	28.8 (24.7–32.9)	82.6 (79.9–85.3)	< 0.001
ALB*HDL	0.625 (0.587–0.663)	63.1 (63.1–63.2)	55.6 (49.6–61.5)	65.2 (62.2–68.2)	30.8 (26.7–34.9)	84.1 (81.4–86.7)	< 0.001
GLU	0.719 (0.685–0.753)	63.7 (63.7–63.7)	74.6 (69.4–79.8)	60.6 (57.6–63.7)	34.5 (30.7–38.4)	89.6 (87.2–91.9)	< 0.001
D-dimer	0.623 (0.587–0.660)	52.1 (52.0–52.1)	75.7 (70.6–80.9)	45.5 (42.3–48.6)	27.9 (24.6–31.1)	87.1 (84.1–90.0)	< 0.001
ALT	0.561 (0.522–0.599)	62.6 (62.6–62.7)	40.3 (34.4–46.2)	68.8 (65.9–71.8)	26.5 (22.2–30.8)	80.6 (77.9–83.3)	< 0.001
ALB	0.643 (0.607–0.680)	55.6 (55.6–55.7)	71.3 (65.9–76.7)	51.3 (48.1–54.5)	28.9 (25.5–32.4)	86.5 (83.7–89.3)	< 0.001
HDL	0.579 (0.540–0.617)	58.6 (58.5–58.6)	54.5 (48.5–60.4)	59.7 (56.6–62.8)	27.3 (23.6–31.1)	82.5 (79.7–85.3)	< 0.001

GLU, glucose; ALT, alanine aminotransferase; ALB, albumin; HDL, high-density lipoprotein; CI, confidence interval; AUC, area under the curve; ACC, accuracy; SEN, sensitivity; SPE, specificity; PPV, positive predictive value; NPV, negative predictive value.

^&^A comparison of AUC was performed using the DeLong test.

**FIGURE 4:**
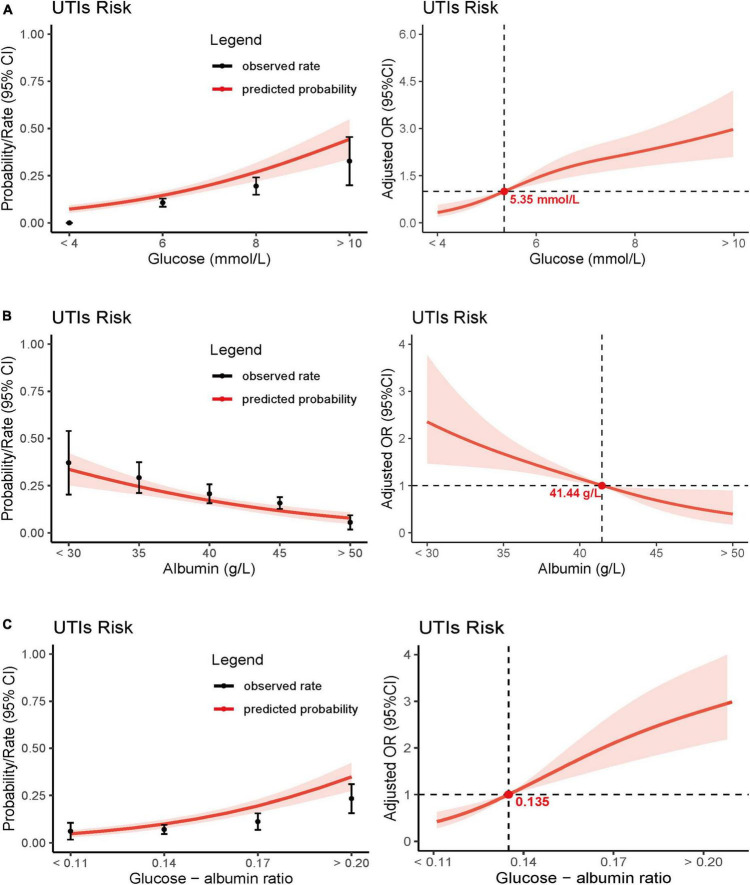
Predicted probabilities and the observed rate of postoperative UTIs: glucose **(A)**, albumin **(B)**, and glucose - albumin ratio **(C)**. Adjusted odds ratio (OR) for postoperative UTIs according to levels of glucose **(A)**, albumin **(B)**, and glucose - albumin ratio **(C)** on a continuous scale.

The correlation between GAR levels and postoperative UTIs in elderly hip fracture patients was assessed, and the results are displayed in Table 3. In a univariate regression analysis, a high GAR level was found to be associated with postoperative UTIs (OR 5.34, 95% CI 3.96–7.22). Even after adjusting for confounding factors (age, gender, smoking, alcohol, hypertension, diabetes mellitus, cardiovascular disease, stroke, chronic kidney disease, prostatic hyperplasia, urolithiasis, vesicoureteral disease, type of hip fracture, ASA classification, surgical modality, indwelling catheterization, indwelling catheterization time, surgery method, and bed rest time), this correlation remained significant (OR 3.20, 95% CI 2.23–4.58). When analyzed as a continuous variable, each 1-SD increase in GAR resulted in a corrected OR of 1.81 (95% CI 1.51–2.17; Table 3) for postoperative UTIs. Furthermore, when GAR levels were examined in quartiles, patients in Q2 (0.14–0.17; OR 2.11, 95% CI 1.07–4.15), Q3 (0.17–0.21; OR 3.36, 95% CI 1.74–6.52), and Q4 (> 0.21; OR 7.55, 95% CI 3.84–14.83) demonstrated significantly higher odds of developing UTIs, compared to patients in Q1 (< 0.14). Notably, a dose-response relationship between GAR levels and postoperative UTIs was observed (*p* for trend < 0.001; Table 3 and Figure 4).

**TABLE 3:** Unadjusted and adjusted associations between postoperative UTIs and GLU-ALB ratio based on different cut-off values.

Biomarker	Group		Unadjusted OR (95% CI)	*P*	Multivariable regression adjusted OR (95% CI)	*P*	PSM adjusted OR (95% CI)	*P*
Glucose	Continuous	Per SD	1.96 (1.71–2.25)	< 0.001	1.71 (1.44–2.04)	< 0.001	NA	NA
	Best cutoff^&^	< 6.25	1 [Reference]	< 0.001	1 [Reference]	< 0.001	1 [Reference]	< 0.001
		≥ 6.25	4.53 (3.34–6.14)	< 0.001*	2.96 (2.07–4.23)	< 0.001*	2.51 (1.75–3.61)	< 0.001*
	Quartile	Q1 (< 5.40)	1 [Reference]		1 [Reference]		1 [Reference]	
		Q2 (5.40–6.10)	2.03 (1.19–3.48)		1.71 (0.96–3.07)		1.39 (0.88–2.20)	
		Q3 (6.10–7.50)	4.43 (2.67–7.33)		3.01 (1.74–5.21)		2.79 (1.81–4.29)	
		Q4 (> 7.50)	8.80 (5.39–14.37)		4.83 (2.72–8.56)		4.45 (2.81–7.04)	
Albumin	Continuous	Per SD	1.62 (1.41–1.86)	< 0.001	1.37 (1.15–1.62)	< 0.001	NA	NA
	Best cutoff^&^	≥ 38.07	1 [Reference]	< 0.001	1 [Reference]	< 0.01	1 [Reference]	< 0.01
		< 38.07	2.61 (1.95–3.50)		1.71 (1.21–2.43)		1.68 (1.17–2.39)	
	Quartile	Q1 (> 41.00)	1 [Reference]	< 0.001*	1 [Reference]	< 0.001*	1 [Reference]	< 0.001*
		Q2 (38.00–41.00)	1.41 (0.86–2.31)		1.23 (0.71–2.14)		1.03 (0.66–1.61)	
		Q3 (35.00–38.00)	2.78 (1.78–4.33)		1.70 (1.02–2.83)		1.65 (1.08–2.53)	
		Q4 (< 35.00)	3.91 (2.46–6.24)		2.49 (1.44–4.32)		2.51 (1.59–3.97)	
GLU-ALB ratio	Continuous	Per SD	2.16 (1.87–2.51)	< 0.001	1.81 (1.51–2.17)	< 0.001	NA	NA
	Best cutoff^&^	< 0.18	1 [Reference]	< 0.001	1 [Reference]	< 0.001	1 [Reference]	< 0.001
		≥ 0.18	5.34 (3.96–7.22)		3.20 (2.23–4.58)		2.44 (1.69–3.54)	
	Quartile	Q1 (< 0.14)	1 [Reference]	< 0.001*	1 [Reference]	< 0.001*	1 [Reference]	< 0.001*
		Q2 (0.14–0.17)	3.00 (1.58–5.69)		2.11 (1.07–4.15)		1.66 (0.94–2.92)	
		Q3 (0.17–0.21)	6.28 (3.39–11.64)		3.36 (1.74–6.52)		3.85 (2.25–6.58)	
		Q4 (> 0.21)	15.94 (8.75–29.03)		7.55 (3.84–14.83)		8.19 (4.68–14.31)	

SD, standard deviation; NA, not available; OR, odds ratio; PSM, propensity scores matching;

*p for trend;

^&^The best cutoff values for glucose, albumin, and GLU-ALB ratio were determined using Youden’s index.

Moreover, we conducted separate analyses for the correlation between blood glucose levels and albumin levels with postoperative UTIs. Results indicated that both of these biomarkers were independently associated with postoperative UTIs, and a dose-response relationship was evident (*p* for trend < 0.001; Table 3 and Figure 4). For comprehensive details, the multiple regression analyses for the three biomarkers are presented in Supplementary Tables 1, 3, 5 of the Data Supplementary. The E-value for the association between GAR and postoperative UTIs was calculated to be 2.98, with a lower limit of 2.35.

As part of a sensitivity analysis, propensity score matching was conducted. Following the matching process, all covariates exhibited balanced differences (SMD < 0.1, see Supplementary Tables 2, 4, 6). Importantly, the propensity-matched analysis confirmed the significant correlation between GAR and postoperative UTIs (OR 2.44, 95% CI 1.69–3.54; Table 3), thus enhancing the robustness of the findings.

## Discussion

This retrospective cohort study aimed to assess the prognostic value of different biomarker combinations in predicting postoperative UTIs among elderly patients with hip fractures. Our findings indicate that a high GAR with a threshold of 0.18 is significantly associated with an increased risk of postoperative UTIs. Among the tested biomarker combinations, GAR demonstrated the highest predictive accuracy for postoperative UTIs.

Urinary tract infections (UTIs) consistently present as significant complications in diabetic patients, a trend supported by numerous studies (19–21). Diabetes, a chronic illness, affects various organ systems within the human body and often leads to complications, including infections (35, 36). Several mechanisms contribute to the development of UTIs within this demographic (20, 37, 38). A hyperglycemic environment intrinsic to diabetic patients hinders their immune response. This inhibition underscores the increased invasiveness and proliferation of pathogens whilst also curtailing the secretion of critical cytokines, such as interleukins (20, 37). Hyperglycemia compromises the functions of leukocytes, granulocytes, and macrophages, thereby expediting bacterial growth in glucose-rich environments (38). An in vitro study illustrated faster bacterial growth in urine containing a high-glucose level than in glucose-free human urine (39). Additionally, a recent pivotal study by Murtha et al. (40) suggested that lowered insulin receptor expression coupled with restricted insulin signaling in diabetic patients could significantly impact the immune system’s innate function in the urothelium and renal epithelium, thus heightening the proneness to UTIs.

Prior research has extensively examined the correlation between diabetic patients and UTI, although the influence of admission hyperglycemia levels on acute trauma patients’ susceptibility to UTI remains under-discussed (18, 41). This study endeavored to explain the connection between post-traumatic admission hyperglycemia and UTIs in patients with hip fractures. The findings pointed to a direct relation between admission hyperglycemia and increased UTI probability, demonstrating a dose-response relationship. Here, rising glucose levels were invariably tied to an amplified risk of infection. Several recent studies suggested post-traumatic injuries provoke specific autonomic and endocrine reactions, raising catecholamine and glycogen levels that potentially harm immune function (42, 43). Moreover, Karunakar and Staples (41) indicated stress-induced hyperglycemia as a significant risk factor for infectious complications in orthopedic trauma patients, including those without a diabetes history.

Recent years have witnessed an increasing utilization of serum albumin levels in hip fracture patients, demonstrating their association with perioperative complications and long-term postoperative mortality (24, 25). Our research indicated a connection between hypoalbuminemia at the time of admission and a rise in the likelihood of urinary tract infections, exhibiting a dose-response relationship where lower albumin levels corresponded with a heightened risk of infection. Commonly, hypoalbuminemia is viewed as a manifestation of malnutrition (22, 23, 26). Emerging evidence suggests that the significant decline in serum albumin levels in hip fracture patients might be attributed to inflammation, rather than pre-existing malnutrition (26, 44). Albumin, exclusively synthesized by the liver, represents the most abundant plasma protein. However, the comprehensive understanding of its metabolic functions remains incomplete (45). Apart from its established role in maintaining fluid-electrolyte balance, albumin may possess immunomodulatory functions (46). Circulating albumin interacts with various inflammatory mediators, promoting neutrophil degranulation and enhancing phagocytosis (47). Consequently, diminished serum albumin levels can impair immune system efficiency, resulting in an increased susceptibility to infectious complications (48). Furthermore, hypoalbuminemia serves as a straightforward indicator of malnutrition, which is a prominent cause of immune response impairment and a robust predictor of hospital-acquired infections (49).

While the novel combination of glucose and albumin ratio (GAR) identified in this study introduces new insight, previous literature has extensively discussed the relationship between glucose, albumin, and UTIs (14, 50–52). A meta-analysis by Huang et al. (50), reviewing 183,588 stroke patients, revealed that high glucose levels were linked to a higher likelihood of developing UTIs (OR 2.53, 95% CI 1.45–4.42). Similarly, Shanks et al. (51) demonstrated that intraoperative glucose levels exceeding 8.3 mmol/L were linked to an increased risk of postoperative infections, based on a study involving 3,150 patients undergoing non-cardiac surgery. In a study of 286 elderly patients with UTIs, Kitano et al. (14) observed that hypoalbuminemia significantly correlated with UTI development. Furthermore, Tal et al. (52) discovered a strong association between low serum albumin levels and mortality in elderly patients with UTIs (*p* < 0.002). Nonetheless, the relationship between GAR and postoperative UTIs is intricate, potentially confounded by various factors. Thus, further investigations are necessary to explore this potential association thoroughly and determine the optimal utilization of GAR as a predictor of postoperative UTIs following hip fracture.

Glucose and albumin, widely used biomarkers in clinical settings, provide a cost-effective and readily available approach to identifying high-risk patients. Importantly, both biomarkers can be modified, enabling physicians to take proactive interventions. Currently, researchers are exploring the management of postoperative UTIs in hip patients and recognizing the significance of controlling UTIs through insulin or hypoglycemic agents in combination with antimicrobial therapy (53, 54). Simultaneously, early nutritional optimization may enhance patient prognosis by providing supplemental calories and protein, modulating immune function, and maintaining normal cellular metabolism (55). Preoperative nutritional intervention has been shown to significantly improve the prognosis of infection-related complications following fracture surgery (56). Additionally, preoperative oral nutritional supplementation (ONS) has demonstrated its preventive effects on exacerbating complications in elderly patients undergoing hip fracture surgery (57). Consequently, when appropriately selected, prophylactic use of antibiotics, hypoglycemic agents, and early nutritional optimization can effectively reduce UTI incidence and overall hospital stay (Supplementary Figures 3–5).

Several limitations should be noted in this study. Firstly, we only examined basic hematological biomarkers commonly used in clinical practice. It is possible that more complex combinations may serve as more reliable indicators of poor prognosis than our newly developed GAR. Secondly, this study is based on a retrospective analysis conducted in a single-center setting. While the robustness of the results is supported by a large sample size and sophisticated statistical methods, the applicability and generalizability of GAR to other institutions need to be validated through large sample sizes from diverse institutions. Thirdly, blood glucose and albumin levels can fluctuate during hospitalization. Though we only considered baseline blood glucose and albumin levels recorded at admission to mitigate confounding effects, we did not assess these relationships over time. Lastly, it is essential to acknowledge that our study spans a data collection period of ten years, a factor that could potentially introduce unquantifiable influences on our research outcomes. In maintaining scientific integrity, it is imperative to transparently disclose this confounding variable.

## Conclusion

This extensive study has identified GAR as a direct, synergistic, and readily available biomarker that predicts the risk of postoperative urinary tract infections (UTIs) in elderly patients with hip fractures. The analysis shows that GAR, obtained upon admission, enables the stratification of patients who are at a high risk of developing postoperative UTIs. Subsequent clinical trials should be carried out to evaluate the utility of this biomarker in managing hip.

## Data availability statement

The original contributions presented in this study are included in the article/Supplementary material, further inquiries can be directed to the corresponding author.

## Ethics statement

The studies involving humans were approved by the Institutional Review Board of the Ethics Committee of Dandong Central Hospital. The studies were conducted in accordance with the local legislation and institutional requirements. The Ethics Committee/Institutional Review Board waived the requirement of written informed consent for participation from the participants or the participants’ legal guardians/next of kin because this study was approved by the Ethics Committees of Dandong Central Hospital (No. DDZX-20230711) and conducted in accordance with the ethical principles of the Helsinki Declaration of 1964 and its later revisions. The Ethics Committee sought and obtained a waiver of consent for this cohort study.

## Author contributions

WW: Data curation, Formal analysis, Investigation, Methodology, Project administration, Software, Writing – original draft. WT: Formal analysis, Investigation, Methodology, Software, Writing – original draft. WY: Formal analysis, Investigation, Methodology, Project administration, Resources, Software, Writing – original draft, Writing – review & editing. QL: Conceptualization, Data curation, Resources, Software, Supervision, Validation, Visualization, Writing – review & editing. WD: Conceptualization, Funding acquisition, Supervision, Validation, Visualization, Writing – review & editing.
