# Supplementary material for: Glucose-albumin ratio (GAR) as a novel biomarker of postoperative urinary tract infection in elderly hip fracture patients
Source: Front Med (Lausanne). 2024 Jul 15;11:1366012. doi: 10.3389/fmed.2024.1366012 (PMC11284060; doi:10.3389/fmed.2024.1366012)
Supplement: Supplementary file 1 [file Data_Sheet_1.pdf]

Appendix:

eFigure1 Distribution of markers and their combinations in the urinary tract infections and non-urinary tract infections groups

eFigure2 ROC curves of each marker for Postoperative UTIs.

eFigure3 Diagnostic process schematic for perioperative hyperglycemic patients.

eFigure4 Schematic of the treatment process for perioperative hyperglycemic patients.

eFigure5 Schematic of the treatment process for patients with perioperative malnutrition.

eTable1 Multivariate Analysis for postoperative UTIs (Glucose).

eTable2 Patient Characteristics before and After Propensity Score Matching by best cutoff value of Glucose.

eTable3 Multivariate Analysis for postoperative UTIs (Albumin).

eTable4 Patient Characteristics before and After Propensity Score Matching by best cutoff value of Albumin.

eTable5 Multivariate Analysis for postoperative UTIs (GLU-ALB ratio).

eTable6 Patient Characteristics before and After Propensity Score Matching by best cutoff value of GLU-ALB ratio (GAR).

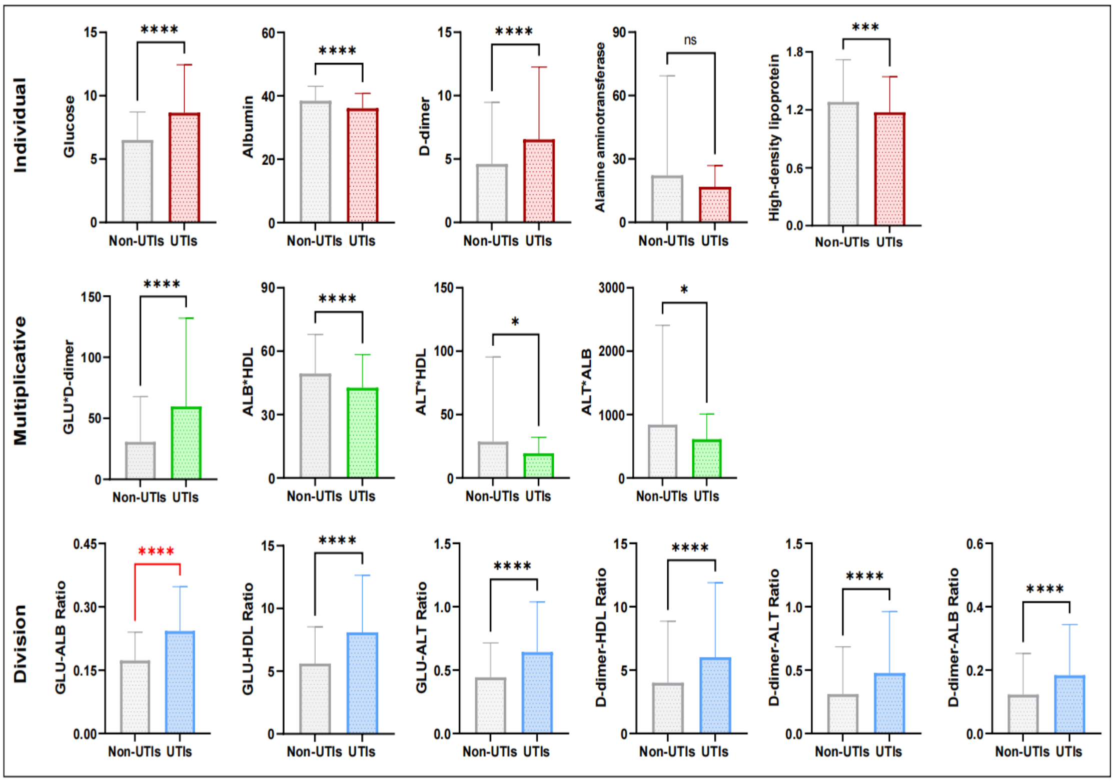

eFigure1 Distribution of markers and their combinations in the urinary tract infections and non-urinary tract infections groups.

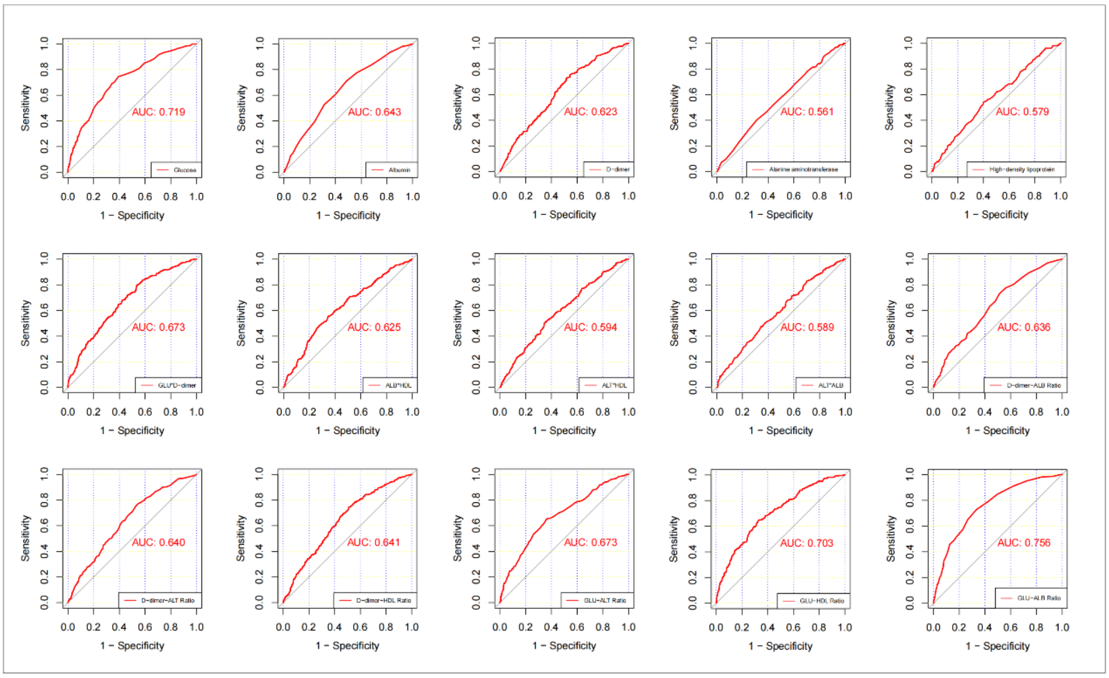

eFigure2 ROC curves of each marker for Postoperative UTIs.

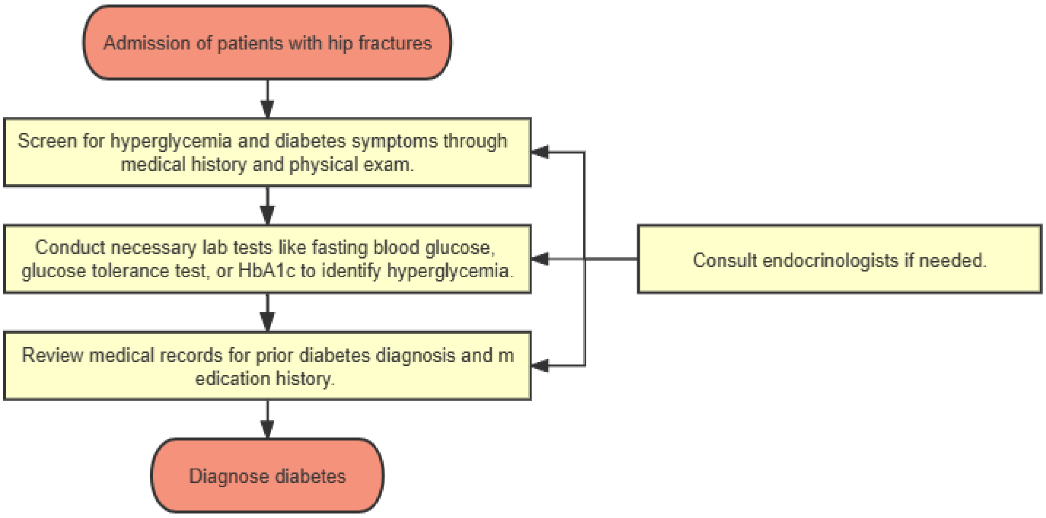

eFigure3 Diagnostic process schematic for perioperative hyperglycemic patients.

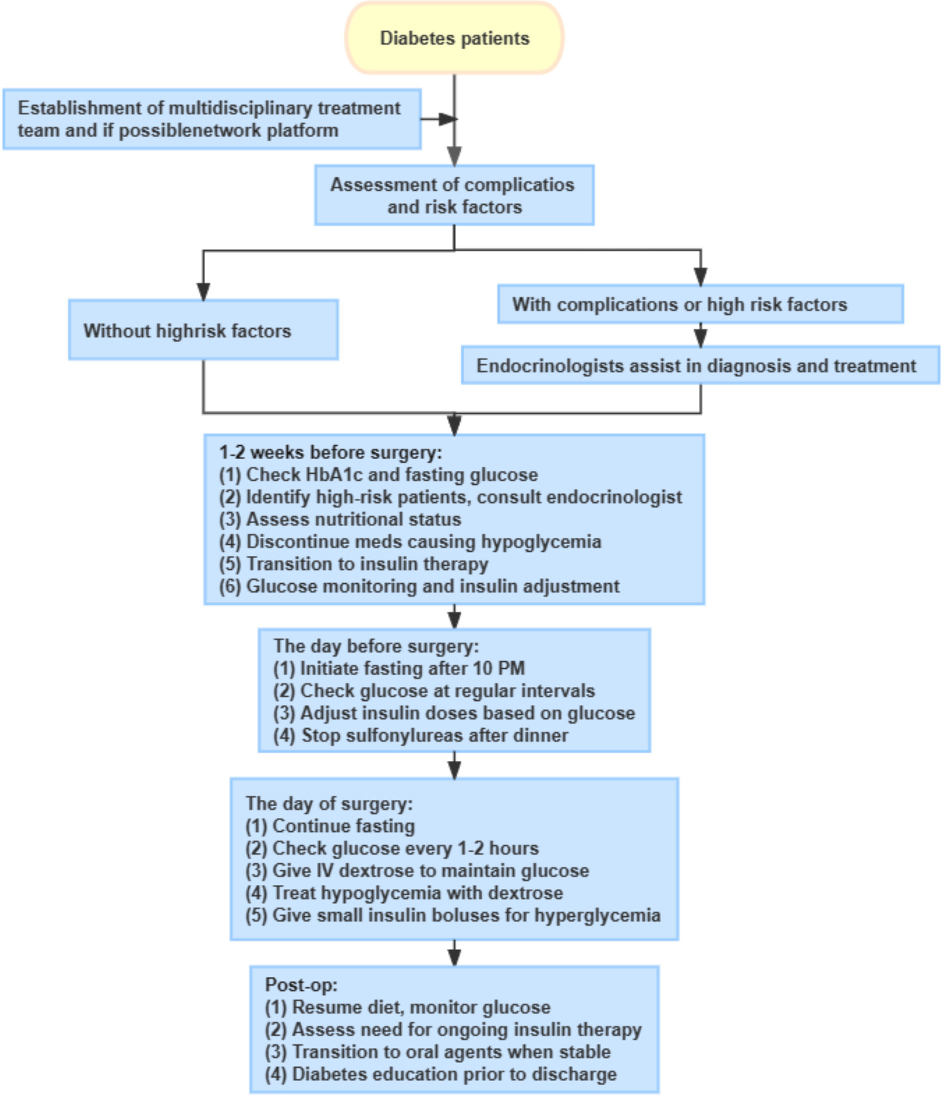

eFigure4 Schematic of the treatment process for perioperative hyperglycemic patients.

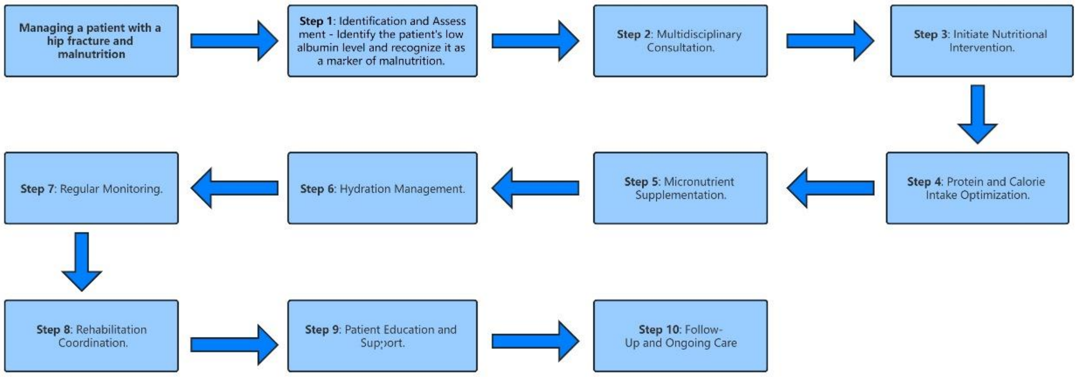

eFigure5 Schematic of the treatment process for patients with perioperative malnutrition.

eTable1 Multivariate Analysis for postoperative UTIs (Glucose).

Characteristics	Univariate			Multivariate		
	OR	95%CI	P-value	OR	95%CI	P-value
Demographic						
Age, × years	1.05	1.04-1.07	<0.001	1.03	1.01-1.05	0.004
Female gender	2.29	1.69-3.11	<0.001	2.60	1.72-3.95	<0.001
Smoking	0.59	0.39-0.88	0.01	1.33	0.75-2.36	0.33
Alcohol	0.52	0.32-0.86	0.01	0.91	0.44-1.90	0.80
Comorbidities						
Hypertension	2.19	1.65-2.90	<0.001	1.26	0.89-1.77	0.20
Diabetes	3.07	2.29-4.11	<0.001	1.80	1.27-2.57	0.001
Cardiovascular disease	1.66	1.25-2.20	<0.001	1.01	0.72-1.43	0.95
Stroke	1.49	1.11-2.00	0.01	1.11	0.78-1.58	0.55
Chronic kidney disease	3.69	2.21-6.14	<0.001	3.05	1.57-5.93	0.001
Prostate hyperplasia	2.29	1.03-5.12	0.04	2.79	1.03-7.60	0.05
Urolithiasis	2.66	1.06-6.69	0.04	0.46	0.14-1.53	0.21
Vesicoureteral disease	4.05	2.37-6.94	<0.001	2.17	1.05-4.51	0.04
Neoplasms	0.67	0.39-1.12	0.13	<NA>	<NA>	<NA>
Operation						
Fracture type	0.56	0.45-0.70	<0.001	0.44	0.32-0.63	<0.001
ASA Classification	1.54	1.16-2.04	0.003	0.73	0.52-1.03	0.08
Surgery method	0.87	0.77-0.97	0.01	0.77	0.63-0.94	0.01
Catheterization	2.44	1.85-3.23	<0.001	1.19	0.80-1.77	0.40
Indwelling catheter time	1.21	1.16-1.27	<0.001	1.15	1.09-1.22	<0.001
Intraoperative time	1.15	0.98-1.35	0.08	1.08	0.89-1.32	0.45
Bedridden time	1.08	1.04-1.11	<0.001	1.04	1.00-1.08	0.05
Laboratory findings						
Glucose*	4.53	3.34-6.14	<0.001	2.96	2.07-4.23	<0.001

*cutoff= 6.25, which was identified by Youden's index.

eTable2 Patient Characteristics before and After Propensity Score Matching by best cutoff value of Glucose.

Characteristics	Before matching			After matching		
	Glu < 6.25 (n=652)	Glu ≥ 6.25 (n=579)	SMD	Glu < 6.25 (n=373)	Glu ≥ 6.25 (n=373)	SMD
Demographic						
Age, × years (Mean, SD)	73.25 (9.57)	76.96 (8.98)	0.40	76.80 (9.18)	76.79 (9.51)	0.001
Female gender (n, %)	363 (55.67%)	378 (65.28%)	0.20	221 (59.25%)	227 (60.86%)	0.03
Smoking (n, %)	125 (19.17%)	88 (15.20%)	0.11	67 (17.96%)	63 (16.89%)	0.03
Alcohol (n, %)	84 (12.88%)	58 (10.02%)	0.09	42 (11.26%)	43 (11.53%)	0.01
Comorbidities						
Hypertension (n, %)	270 (41.41%)	355 (61.31%)	0.41	221 (59.25%)	212 (56.84%)	0.05
Diabetes (n, %)	50 (7.67%)	235 (40.59%)	0.83	50 (13.40%)	54 (14.48%)	0.03
Cardiovascular disease (n, %)	174 (26.69%)	204 (35.23%)	0.19	118 (31.64%)	122 (32.71%)	0.02
Stroke (n, %)	148 (22.70%)	178 (30.74%)	0.18	106 (28.42%)	101 (27.08%)	0.03
Chronic kidney disease (n, %)	32 (4.91%)	32 (5.53%)	0.03	23 (6.17%)	23 (6.17%)	<0.001
Prostate hyperplasia (n, %)	10 (1.53%)	16 (2.76%)	0.09	10 (2.68%)	8 (2.14%)	0.04
Urolithiasis (n, %)	10 (1.53%)	9 (1.55%)	0.002	7 (1.88%)	7 (1.88%)	<0.001
Vesicoureteral disease (n, %)	26 (3.99%)	31 (5.35%)	0.07	19 (5.09%)	19 (5.09%)	<0.001
Neoplasms (n, %)	56 (8.59%)	56 (9.67%)	0.04	39 (10.46%)	34 (9.12%)	0.05
Operation						
Fracture type						
Femoral neck fracture (n, %)	389 (59.66%)	250 (43.18%)	0.31	170 (45.58%)	170 (45.58%)	0.004
Intertrochanteric fracture (n, %)	231 (35.43%)	290 (50.09%)		176 (47.18%)	177 (47.45%)	
Subtrochanteric fracture (n, %)	32 (4.91%)	39 (6.74%)		27 (7.24%)	26 (6.97%)	
ASA Classification						
III-IV (n, %)	330 (50.61%)	370 (63.90%)	0.27	237 (63.54%)	228 (61.13%)	0.05
I-II (n, %)	322 (49.39%)	209 (36.10%)		136 (36.46%)	145 (38.87%)	
Surgery method						
Total Hip Arthroplasty (n, %)	97 (14.88%)	56 (9.67%)		40 (10.72%)	33 (8.85%)	
Hemiarthroplasty (n, %)	161 (24.69%)	150 (25.91%)		99 (26.54%)	102 (27.35%)	
Intramedullary nail fixation (n, %)	181 (27.76%)	232 (40.07%)	0.11	137 (36.73%)	136 (36.46%)	0.03
Internal fixation with steel plate (n, %)	72 (11.04%)	95 (16.41%)		55 (14.75%)	64 (17.16%)	
Internal fixation with hollow nails (n, %)	141 (21.63%)	46 (7.94%)		42 (11.26%)	38 (10.19%)	
Catheterization (n, %)	288 (44.17%)	282 (48.70%)	0.09	174 (46.65%)	184 (49.33%)	0.05
Indwelling catheter time, ×days (Mean, SD)	1.45 (2.66)	2.18 (4.17)	0.21	1.82 (3.19)	1.99 (3.26)	0.05
Intraoperative time, ×hours (Mean, SD)	1.63 (0.76)	1.70 (0.86)	0.09	1.68 (0.79)	1.72 (0.94)	0.05
Bedridden time, ×days (Mean, SD)	5.40 (3.36)	6.44 (4.63)	0.26	5.90 (3.74)	5.85 (4.32)	0.01

eTable3 Multivariate Analysis for postoperative UTIs (Albumin).

Characteristics	Univariate			Multivariate		
	OR	95%CI	P-value	OR	95%CI	P-value
Demographic						
Age, × years	1.05	1.04-1.07	<0.001	1.03	1.01-1.05	0.01
Female gender	2.29	1.69-3.11	<0.001	2.86	1.89-4.31	<0.001
Smoking	0.59	0.39-0.88	0.01	1.29	0.73-2.27	0.38
Alcohol	0.52	0.32-0.86	0.01	1.02	0.50-2.10	0.95
Comorbidities						
Hypertension	2.19	1.65-2.90	<0.001	1.37	0.98-1.92	0.07
Diabetes	3.07	2.29-4.11	<0.001	2.69	1.92-3.77	<0.001
Cardiovascular disease	1.66	1.25-2.20	<0.001	0.98	0.70-1.37	0.91
Stroke	1.49	1.11-2.00	0.01	1.13	0.80-1.60	0.48
Chronic kidney disease	3.69	2.21-6.14	<0.001	2.76	1.45-5.25	0.002
Prostate hyperplasia	2.29	1.03-5.12	0.04	3.37	1.28-8.92	0.01
Urolithiasis	2.66	1.06-6.69	0.04	0.39	0.12-1.27	0.12
Vesicoureteral disease	4.05	2.37-6.94	<0.001	2.05	1.00-4.19	0.05
Neoplasms	0.67	0.39-1.12	0.13	<NA>	<NA>	<NA>
Operation						
Fracture type	0.56	0.45-0.70	<0.001	0.45	0.32-0.63	<0.001
ASA Classification	1.54	1.16-2.04	0.003	0.71	0.51-1.01	0.05
Surgery method	0.87	0.77-0.97	0.01	0.79	0.65-0.95	0.02
Catheterization	2.44	1.85-3.23	<0.001	1.16	0.78-1.71	0.47
Indwelling catheter time	1.21	1.16-1.27	<0.001	1.15	1.08-1.22	<0.001
Intraoperative time	1.15	0.98-1.35	0.08	1.11	0.92-1.35	0.28
Bedridden time	1.08	1.04-1.11	<0.001	1.04	1.01-1.08	0.03
Laboratory findings						
Albumin[*]	2.61	1.95-3.50	<0.001	1.71	1.21-2.43	0.002

*cutoff= 38.07, which was identified by Youden's index.

eTable4 Patient Characteristics before and After Propensity Score Matching by best cutoff value of Albumin.

Characteristics	Before matching			After matching		
	Alb ≥ 38.07 (n=571)	Alb < 38.07 (n=660)	SMD	Alb ≥ 38.07 (n=384)	Alb < 38.07 (n=384)	SMD
Demographic						
Age, × years (Mean, SD)	71.08 (8.38)	78.38 (9.06)	0.84	74.17 (8.18)	74.33 (8.55)	0.02
Female gender (n, %)	344 (60.25%)	397 (60.15%)	0.002	234 (60.94%)	235 (61.20%)	0.01
Smoking (n, %)	115 (20.14%)	98 (14.85%)	0.14	65 (16.93%)	70 (18.23%)	0.03
Alcohol (n, %)	80 (14.01%)	62 (9.39%)	0.14	44 (11.46%)	43 (11.20%)	0.01
Comorbidities						
Hypertension (n, %)	244 (42.73%)	381 (57.73%)	0.30	200 (52.08%)	202 (52.60%)	0.01
Diabetes (n, %)	125 (21.89%)	160 (24.24%)	0.06	93 (24.22%)	96 (25.00%)	0.02
Cardiovascular disease (n, %)	134 (23.47%)	244 (36.97%)	0.30	118 (30.73%)	126 (32.81%)	0.05
Stroke (n, %)	118 (20.67%)	208 (31.52%)	0.25	95 (24.74%)	98 (25.52%)	0.02
Chronic kidney disease (n, %)	20 (3.50%)	44 (6.67%)	0.14	17 (4.43%)	20 (5.21%)	0.04
Prostate hyperplasia (n, %)	7 (1.23%)	19 (2.88%)	0.12	7 (1.82%)	7 (1.82%)	<0.001
Urolithiasis (n, %)	5 (0.88%)	14 (2.12%)	0.10	5 (1.30%)	4 (1.04%)	0.02
Vesicoureteral disease (n, %)	16 (2.80%)	41 (6.21%)	0.17	15 (3.91%)	18 (4.69%)	0.04
Neoplasms (n, %)	51 (8.93%)	61 (9.24%)	0.01	32 (8.33%)	33 (8.59%)	0.01
Operation						
Fracture type			0.39			0.02
Femoral neck fracture (n, %)	367 (64.27%)	272 (41.21%)		212 (55.21%)	206 (53.65%)	
Intertrochanteric fracture (n, %)	172 (30.12%)	349 (52.88%)		143 (37.24%)	159 (41.41%)	
Subtrochanteric fracture (n, %)	32 (5.60%)	39 (5.91%)		29 (7.55%)	19 (4.95%)	
ASA Classification			0.43			0.01
III-IV (n, %)	261 (45.71%)	439 (66.52%)		226 (58.85%)	227 (59.11%)	
I-II (n, %)	310 (54.29%)	221 (33.48%)		158 (41.15%)	157 (40.89%)	
Surgery method			0.12			0.04
Total Hip Arthroplasty (n, %)	91 (15.94%)	62 (9.39%)		52 (13.54%)	53 (13.80%)	
Hemiarthroplasty (n, %)	143 (25.04%)	168 (25.45%)		125 (32.55%)	110 (28.65%)	
Intramedullary nail fixation (n, %)	139 (24.34%)	274 (41.52%)		114 (29.69%)	123 (32.03%)	
Internal fixation with steel plate (n, %)	62 (10.86%)	105 (15.91%)		52 (13.54%)	55 (14.32%)	
Internal fixation with hollow nails (n, %)	136 (23.82%)	51 (7.73%)		41 (10.68%)	43 (11.20%)	
Catheterization (n, %)	232 (40.63%)	338 (51.21%)	0.21	174 (45.31%)	184 (47.92%)	0.05
Indwelling catheter time, ×days (Mean, SD)	1.27 (2.68)	2.24 (3.98)	0.29	1.52 (3.09)	1.74 (3.06)	0.07
Intraoperative time, ×hours (Mean, SD)	1.66 (0.85)	1.66 (0.77)	0.003	1.73 (0.92)	1.66 (0.77)	0.08
Bedridden time, ×days (Mean, SD)	5.37 (3.49)	6.33 (4.41)	0.24	5.89 (3.80)	5.72 (3.09)	0.05

eTable5 Multivariate Analysis for postoperative UTIs (GLU-ALB ratio).

Characteristics	Univariate			Multivariate		
	OR	95%CI	P-value	OR	95%CI	P-value
Demographic						
Age, × years	1.05	1.04-1.07	<0.001	1.02	1.00-1.04	0.03
Female gender	2.29	1.69-3.11	<0.001	2.67	1.76-4.06	<0.001
Smoking	0.59	0.39-0.88	0.01	1.27	0.72-2.26	0.41
Alcohol	0.52	0.32-0.86	0.01	1.04	0.50-2.17	0.91
Comorbidities						
Hypertension	2.19	1.65-2.90	<0.001	1.27	0.90-1.80	0.17
Diabetes	3.07	2.29-4.11	<0.001	1.72	1.20-2.47	0.003
Cardiovascular disease	1.66	1.25-2.20	<0.001	0.98	0.70-1.38	0.91
Stroke	1.49	1.11-2.00	0.01	1.10	0.77-1.56	0.61
Chronic kidney disease	3.69	2.21-6.14	<0.001	2.74	1.41-5.32	0.003
Prostate hyperplasia	2.29	1.03-5.12	0.04	3.37	1.24-9.19	0.02
Urolithiasis	2.66	1.06-6.69	0.04	0.41	0.12-1.40	0.16
Vesicoureteral disease	4.05	2.37-6.94	<0.001	2.24	1.07-4.68	0.03
Neoplasms	0.67	0.39-1.12	0.13	<NA>	<NA>	<NA>
Operation						
Fracture type	0.56	0.45-0.70	<0.001	0.46	0.33-0.65	<0.001
ASA Classification	1.54	1.16-2.04	0.003	0.72	0.51-1.02	0.06
Surgery method	0.87	0.77-0.97	0.01	0.77	0.63-0.93	0.01
Catheterization	2.44	1.85-3.23	<0.001	1.21	0.81-1.81	0.36
Indwelling catheter time	1.21	1.16-1.27	<0.001	1.15	1.09-1.22	<0.001
Intraoperative time	1.15	0.98-1.35	0.08	1.04	0.85-1.27	0.71
Bedridden time	1.08	1.04-1.11	<0.001	1.04	0.99-1.08	0.07
Laboratory findings						
GLU-ALB ratio*	5.34	3.96-7.22	<0.001	3.20	2.23-4.58	<0.001

*cutoff= 0.18, which was identified by Youden's index.

eTable6 Patient Characteristics before and After Propensity Score Matching by best cutoff value of GLU-ALB ratio (GAR).

Characteristics	Before matching			After matching		
	GAR < 0.18 (n=715)	GAR ≥ 0.18 (n=516)	SMD	GAR < 0.18 (n=325)	GAR ≥ 0.18 (n=325)	SMD
Demographic						
Age, × years (Mean, SD)	72.65 (9.30)	78.23 (8.74)	0.62	77.25 (8.97)	77.32 (9.11)	0.01
Female gender (n, %)	401 (56.08%)	340 (65.89%)	0.20	212 (65.23%)	200 (61.54%)	0.07
Smoking (n, %)	142 (19.86%)	71 (13.76%)	0.16	50 (15.38%)	54 (16.62%)	0.03
Alcohol (n, %)	99 (13.85%)	43 (8.33%)	0.18	29 (8.92%)	32 (9.85%)	0.03
Comorbidities						
Hypertension (n, %)	302 (42.24%)	323 (62.60%)	0.42	188 (57.85%)	185 (56.92%)	0.02
Diabetes (n, %)	65 (9.09%)	220 (42.64%)	0.83	63 (19.38%)	66 (20.31%)	0.02
Cardiovascular disease (n, %)	180 (25.17%)	198 (38.37%)	0.29	110 (33.85%)	113 (34.77%)	0.02
Stroke (n, %)	157 (21.96%)	169 (32.75%)	0.24	94 (28.92%)	87 (26.77%)	0.05
Chronic kidney disease (n, %)	29 (4.06%)	35 (6.78%)	0.12	19 (5.85%)	21 (6.46%)	0.03
Prostate hyperplasia (n, %)	14 (1.96%)	12 (2.33%)	0.03	7 (2.15%)	8 (2.46%)	0.02
Urolithiasis (n, %)	9 (1.26%)	10 (1.94%)	0.05	6 (1.85%)	6 (1.85%)	<0.001
Vesicoureteral disease (n, %)	26 (3.64%)	31 (6.01%)	0.11	20 (6.15%)	20 (6.15%)	<0.001
Neoplasms (n, %)	63 (8.81%)	49 (9.50%)	0.02	29 (8.92%)	35 (10.77%)	0.06
Operation						
Fracture type			0.42			0.02
Femoral neck fracture (n, %)	439 (61.40%)	200 (38.76%)		149 (45.85%)	144 (44.31%)	
Intertrochanteric fracture (n, %)	241 (33.71%)	280 (54.26%)		154 (47.38%)	161 (49.54%)	
Subtrochanteric fracture (n, %)	35 (4.90%)	36 (6.98%)		22 (6.77%)	20 (6.15%)	
ASA Classification			0.38			0.02
III-IV (n, %)	352 (49.23%)	348 (67.44%)		209 (64.31%)	206 (63.38%)	
I-II (n, %)	363 (50.77%)	168 (32.56%)		116 (35.69%)	119 (36.62%)	
Surgery method			0.06			0.04
Total Hip Arthroplasty (n, %)	103 (14.41%)	50 (9.69%)		29 (8.92%)	33 (10.15%)	
Hemiarthroplasty (n, %)	195 (27.27%)	116 (22.48%)		100 (30.77%)	85 (26.15%)	
Intramedullary nail fixation (n, %)	188 (26.29%)	225 (43.60%)		117 (36.00%)	120 (36.92%)	
Internal fixation with steel plate (n, %)	79 (11.05%)	88 (17.05%)		47 (14.46%)	57 (17.54%)	
Internal fixation with hollow nails (n, %)	150 (20.98%)	37 (7.17%)		32 (9.85%)	30 (9.23%)	
Catheterization (n, %)	314 (43.92%)	256 (49.61%)	0.11	161 (49.54%)	161 (49.54%)	<0.001
Indwelling catheter time, ×days (Mean, SD)	1.40 (2.55)	2.34 (4.39)	0.26	1.90 (3.18)	1.83 (2.80)	0.02
Intraoperative time, ×hours (Mean, SD)	1.60 (0.73)	1.75 (0.90)	0.18	1.68 (0.78)	1.69 (0.83)	0.02
Bedridden time, ×days (Mean, SD)	5.30 (3.31)	6.69 (4.76)	0.34	5.98 (3.63)	6.00 (3.66)	0.01
